# Medical Convention of Kentucky

**Published:** 1841-02

**Authors:** 


### THE WESTERN JOURNAL.

Vol. III.—No. II.

LOUISVILLE, FEBRUARY 1, 1841.

### MEDICAL CONVENTION OF KENTUCKY,

We have twice referred to a proposed Convention of the physicians of Kentucky, called by the Medical Association of North East Kentucky, to be held at Frankfort, on the first Monday of January. It was held; and we write this notice on board the steam boat, returning from it.

About sixty physicians attended, and their deliberations were characterized by harmony, and a zealous prosecution of the object for which they assembled. That object was the formation of a State Medical Society. To this end a Constitution was reported by the Committee raised for that purpose, and after careful examination adopted, we believe, unanimously. A system of rules and principles of professional intercourse was made out, and prefixed to it; and the corresponding secretary was ordered to transmit a copy of both to the seat of justice of every county of the State, directed to the physicians of the county.

The adoption of these, and the formation of district associations, will make them members of the State Medical Society. Thus the representative principle has not been admitted into this organization. We congratulate the profession of our state on this exclusion, convinced that the meetings of a state society composed of *Delegates* can never be as profitable, as meetings which embrace a large number of the profession. We hope and trust that our brethren throughout the State may so far approve of what will be sent to them, as to co-operate in the formation of the proposed society; and afterwards attend it numerously.

During the sitting of the Convention a committee appointed for that purpose, reported several resolutions on the subject of medical education, and the preparatory acquirements of students, which were unanimously adopted.

Since the above was written we have received a printed copy of the Constitution adopted by the Convention, with a resolution for carrying it into effect which, as interesting to our readers in Kentucky, and, perhaps, in other parts of the West, we here extract:

"This Society shall be known by the name of the *State Medical Society of Kentucky*, and shall convene, annually, on the Wednesday succeeding the second Monday of January, in the town of Frankfort. The President shall call the Society to order at 10 A. M., and the meetings shall continue from day to day, till the business of the Society be concluded.

"*Exercises of the Society.*

"The exercises of the Society shall consist of papers, or addresses, on some subject connected with the science of medicine. A paper shall be required from at least six of the District Societies, which shall be selected by the President. Reports shall be requested from the District Societies generally, upon the diseases, medical topography, statistics, climate, botany, geology, and any peculiarities of their particular region—communications on points of especial interest and importance shall be requested from the aged and learned of the profession throughout the State.

"All Committees shall be appointed by the President, unless the business be of a very extraordinary nature, when the Committee may be selected by ballot.

"When, in the opinion of the President, it shall be considered of sufficient importance to the interests of the Society, he shall have power to appoint a called meeting, and shall require the Corresponding Secretary to advise the Presidents of all District Societies of the fact, at least one month previous to the appointed time.

"Twenty members shall constitute a quorum to do business.

"In all cases of difficulty in the District Societies, appeal may be had to the State Society, whose decision shall be final.

"No rule or regulation of this Society shall be altered, amended or repealed, nor additional regulations adopted at any meeting, but by a vote of three-fourths of the members present.

"No Society now in existence, or which may hereafter be formed, shall be permitted to hold connection with this, which shall not adopt the foregoing system for their government.

"No District Society shall adopt any rule conflicting with the above.

"All who hold membership in the District Societies shall be considered members of the State Society.

"*Resolved*, That the Corresponding Secretary of this Convention, address to the physicians of each county in the State, (directed to the county seats) a copy of the Constitution and by-laws formed by this Convention, accompanied with this resolution, and urge them to organize themselves *immediately*, into District Societies; the limits of which, shall be prescribed by themselves. And that, as early as practicable, they send to him a copy of their Constitution and by-laws, signed by the officers, together with a list of their members—and if their organization be in accordance with the Constitution aforesaid, he shall thereupon enrol their names as members." D.

